# Olanzapine induced DNA methylation changes support the dopamine hypothesis of psychosis

**DOI:** 10.1186/2049-9256-1-19

**Published:** 2013-11-04

**Authors:** Melkaye G Melka, Christina A Castellani, Benjamin I Laufer, Raj N Rajakumar, Richard O’Reilly, Shiva M Singh

**Affiliations:** Department of Biology, Molecular Genetics Unit, Western Science Centre, The University of Western Ontario, London, Ontario N6A 5B7 Canada; Department of Psychiatry, The University of Western Ontario, London, Ontario N6A 5B7 Canada

**Keywords:** Dopamine, Psychosis, Olanzapine, DNA methylation, Epigenetics

## Abstract

**Background:**

The dopamine (DA) hypothesis of schizophrenia proposes the mental illness is caused by excessive transmission of dopamine in selected brain regions. Multiple lines of evidence, including blockage of dopamine receptors by antipsychotic drugs that are used to treat schizophrenia, support the hypothesis. However, the dopamine D2 receptor (*DRD2*) blockade cannot explain some important aspects of the therapeutic effect of antipsychotic drugs. In this study, we hypothesized that antipsychotic drugs could affect the transcription of genes in the DA pathway by altering their epigenetic profile.

**Methods:**

To test this hypothesis, we examined the effect of olanzapine, a commonly used atypical antipsychotic drug, on the DNA methylation status of genes from DA neurotransmission in the brain and liver of rats*.* Genomic DNA isolated from hippocampus, cerebellum, and liver of olanzapine treated (*n* = 2) and control (*n* = 2) rats were analyzed using rat specific methylation arrays.

**Results:**

Our results show that olanzapine causes methylation changes in genes encoding for DA receptors (*dopamine D1 receptor, dopamine D2 receptor* and *dopamine D5 receptor),* a DA transporter *(solute carrier family 18 member 2),* a DA synthesis *(differential display clone 8),* and a DA metabolism *(catechol-O-methyltransferase)*. We assessed a total of 40 genes in the DA pathway and found 19 to be differentially methylated between olanzapine treated and control rats. Most (17/19) genes showed an increase in methylation, in their promoter regions with *in silico* analysis strongly indicating a functional potential to suppress transcription in the brain.

**Conclusion:**

Our results suggest that chronic olanzapine may reduce DA activity by altering gene methylation. It may also explain the delayed therapeutic effect of antipsychotics, which occurs despite rapid dopamine blockade. Furthermore, given the common nature of epigenetic variation, this lends insight into the differential therapeutic response of psychotic patients who display adequate blockage of dopamine receptors.

## Background

The cause of schizophrenia remains unclear, though much evidence suggests that it may be produced by an excess transmission of dopamine (DA) in selected brain regions [1]. The DA hypothesis is supported by a number of observations. First, there is evidence that psychosis is associated with a hyper-dopaminergic state [2, 3]. Second, all antipsychotic drugs block dopamine receptors, particularly D2 receptors [4]. Third, patients with schizophrenia have elevated D2 receptors producing a behavioral hypersensitivity to dopamine [4, 5]. Furthermore, despite the difficulties of replicating genome wide association studies and candidate gene studies in schizophrenia, a number of genes in the dopamine pathway, including dopamine D1 receptor (*DRD1*), dopamine D2 receptor (*DRD2*), dopamine D5 receptor (*DRD5*), differential display clone 8 (*DDC*), catechol-O-methyltransferase (*COMT*) and solute carrier family 18 member 2 (*SLC18A2*), which is also a vesicular monoamine transporter*,* have been identified as associated with schizophrenia [6]*.* These genes have diverse functions, which include dopamine synthesis and release, receptor occupancy, sensitivity of the dopamine receptors, and hyper-response of the receptor-signalling cascade [1]. Because all antipsychotic drugs block the D2 receptor, it is widely believed that the D2 blockade is central to the therapeutic efficacy of antipsychotic drugs. However, several empirical observations seem to be at odds with this assumption. First, while D2 blockade occurs within several hours, it takes days and sometimes weeks for patients to respond to treatment with antipsychotics [7]. Second, many patients show limited or no therapeutic response despite marked blockade of the D2 receptor [8]. Finally, some patients fail to respond to standard doses of a specific antipsychotic drug, which occurs despite adequate D2 blockade, while interestingly the patient will respond to an equivalent (in terms of D2 blockade) of a second drug [9, 10]. These clinical observations are complemented by imaging studies, which show that antipsychotic D2 blockade can only contribute a small percentage of the variation in antipsychotic effectiveness [11]. Epigenetic mechanisms, particularly DNA methylation, which is responsive to environmental factors [12, 13], have the potential to alter the levels of brain molecules and may contribute to psychotic symptoms and cognitive deficits in schizophrenia [14]. Indeed, recent reports have identified methylation differences in some genes implicated in schizophrenia, as reviewed by Dempster et al. [15]. We propose that antipsychotic drugs affect the transcription of genes in the DA pathway by altering their epigenetic profile. We further hypothesize that this alteration contributes to the therapeutic effect of antipsychotic drugs. In order to test this hypothesis, we have designed this study to assess the effect of a therapeutic dose of an antipsychotic drug (olanzapine) on DNA methylation in two neural (hippocampus, cerebellum) and one non-neural (liver) control tissue *in vivo*.

## Methods

The study used a rat model and genome-wide DNA methylation following Methylated DNA Immunoprecipitation (MeDIP). Adult male Sprague–Dawley rats of 12 weeks of age (250 - 300 g) were purchased from Charles River, PQ, Canada. Upon arrival, rats were separated into individual cages and housed in controlled humidity and temperature on a 12-hour light/dark cycle (lights on at 7:00 a.m.). The Institutional Animal Care Committee of the University of Western Ontario had approved all animal-related procedures used in this study following the Canadian and National Institute of Health Guides on animal experimentation. Animals were weighed and divided into two treatment groups with comparable means of weight. Their stress-induced locomotor activity (following a 5 min tail pinch) was recorded for 30 min using an automated open-field activity chamber (San Diego Instruments, San Diego, CA, USA). A computer that detects the disruption of photocell beams recorded the number of beam breaks per five minutes for half an hour as each animal moves, and the average beam breaks/5 min bins was reported. The rats then received injections of olanzapine (Zyprexa, Lilly, IN, USA; 2.5 mg/kg, i.m.; n = 8) or vehicle (phosphate buffered saline (PBS); n = 8) between 1:30 pm and 3:00 pm daily for 19 days. On day 20, eighteen hours after the last olanzapine/vehicle injection, rats were subjected to stress-induced locomotor activity to assess the therapeutic efficacy of chronic olanzapine. Twenty-four hours after they were decapitated without anaesthesia, micro-punches of brain areas or liver samples were collected and flash frozen.

Genomic DNA isolated from hippocampus, cerebellum and liver from olanzapine treated (*n* = 2) and control (*n* = 2) rats were analyzed using rat methylation arrays. The genomic DNA was isolated from olanzapine treated and saline control samples of hippocampus, cerebellum, and liver to analyze DNA methylation using rat methylation arrays. Genomic DNA was isolated from the interphase layer of TRIzol using sodium citrate, followed by ethanol precipitation and purification using the QIAamp® DNA Micro Kit (QIAGEN, Valencia, CA). DNA was then quantified using a NanoDrop ND-1000 spectrophotometer (Thermo Fisher Scientific Inc., Wilmington, DE) and all samples had OD_260_/OD_280_ nm ratios of 1.8–2.0 and OD_260_/OD_230_ nm ratios of 2.0–2.4. The methylated DNA immunoprecipitation (MeDIP), sample labeling, hybridization, and processing were performed at Arraystar Inc. (Rockville, Maryland, USA). Data analysis involved the comparison of differentially enriched regions between drug exposed (E) and control (C) rats, the log_2_-ratio values were averaged and then used to calculate the M’ value [M’ = Average (log_2_ MeDIPE/InputE) - Average(log_2_ MeDIPC/InputC)] for each probe. NimbleScan sliding-window peak-finding algorithm was run on this data to find the differential enrichment peaks (DEP). Using an R script program, a hierarchical clustering analysis was completed. The probe data matrix was obtained by using PeakScores from differentially methylated regions selected by DEP analysis. This analysis used a “PeakScore” ≥2 to define the DEPs, which is equivalent to the average p-value ≤0.01, for all probes within the peak. This analysis deals with a total of 40 candidate genes of the dopaminergic pathway. “Gene List Venn Diagram” was used to assess the distribution of genes affected across tissue types [16]. Transcription factor binding sites of *DRD5* that showed increased methylation in hippocampus, cerebellum and liver were identified using CTCFBSDB 2.0 [17].

## Results

**Figure 1:**
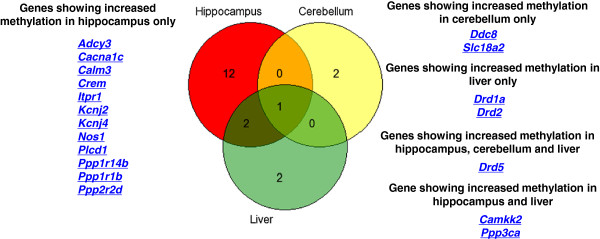
**Genes showing increased methylation in hippocampus, cerebellum and liver, following Olanzapine treatment.** *DRD5* was the only candidate gene that was found to be affected by Olanzapine treatment in both brain regions and the liver.

**Figure 2:**
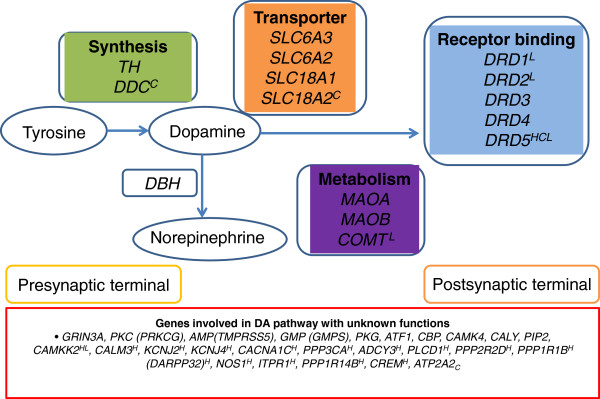
**Candidate genes in the dopaminergic pathway (adapted and modified from Yeh et al.** ***Brain and Cognition 2012;*** **80: 282–289).** Superscripts (H, C and L) represent the genes that showed increased methylation in hippocampus, cerebellum, or liver, respectively. Genes without a superscript were not affected by olanzapine treatment. Subscript (C) represents the gene that showed reduced methylation in the cerebellum.

**Figure 3:**
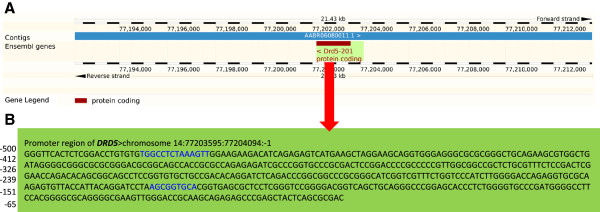
**The genomic location and promoter region of the dopamine D5 receptor gene (** ***DRD5).*** **(A)** Chromosomal position of the *DRD5* (from http://www.ensembl.org/index.html), which showed significantly increased methylation (*p* < 0.01) following olanzapine treatment. **(B)** Sequence of the promoter region of *DRD5,* with CTCF transcription factor binding sites highlighted in **blue** (identified using CTCFBSDB version 2.0 by Bao et al. *Nucleic Acids Research 2008;* 36, D83-D87). The frequency of A/C/G/T in the promoter sequence was 0.174/0.308/0.370/0.148, respectively. The CpG count in the promoter region was 50 out of 250 bp.

Stress-induced locomotor activity was significantly decreased (*p* = 0.001) in olanzapine treated rats 21 days post-stress (32.6 ± 4.4 beam breaks/5 min bins) as compared to that of matched-control (85.6 ± 7.3 beam breaks/5 min bins) (Table 1). Further, Table 2 shows 19 of the 40 genes that were differentially methylated (*p* < 0.01) as a result of olanzapine treatment in the three tissues (hippocampus, cerebellum and liver) studied. The major features of these results are four fold. First, the methylation changes are specific to the promoter regions of the genes. Second, most changes represent an increase in methylation as a result of olanzapine treatment. Third, the methylation changes are tissue (hippocampus, cerebellum, and liver) specific. Fourth and finally, most changes are specific to the hippocampus (Figure 1). For example, 15 of the 19 genes that showed significant increase in methylation are specific to hippocampus of olanzapine treated as compared to the matched control rats (Table 2). Also, 3 out of 40 genes showed significant increase (*DRD5, SLC18A2* and *DDC8*) and one (*ATP2A2*) showed significant decrease in methylation in the cerebellum of olanzapine treated rats (Table 2). Furthermore, 5 of the 40 genes were differentially methylated in olanzapine treated liver as compared to the matched-controls (Table 2). These included the DA receptor genes (*DRD1, DRD2 and DRD5), PPP3CA and CAMKK2*. Also, one gene, C*OMT*, showed decreased methylation in olanzapine treated liver samples. Overall, a number of genes related to DA functioning are differentially methylated as a consequence of olanzapine treatment *in vivo*. They are tissue (hippocampus) specific with only 2 of the 19 genes affected in hippocampus and liver (*PPP3CA and CAMKK2*); with one (*DRD5*) affected in all three tissues in the same direction (Figure 1). Furthermore, *DARPP32*, *ADCY3* (*AC3*), DA receptor genes and a DA transporter gene (*SLC18A2/VMAT2*) were signified in the DA *receptor signalling* pathway amongst genes that showed significant increases in methylation following olanzapine treatment (Figure 2). Also, we assessed gene-specific sequences that were methylated in the olanzapine treated samples. The region of *DRD5* that was differentially methylated following olanzapine treatment has the necessary features of a functioning promoter, as illustrated by the identified CTCF transcription factor binding sites (Figure 3).

**Table 1:** **Locomotor activity (average number of beam breaks/5 minutes ± stderr) of olanzapine treated rats and their matched-controls during day 0 and 21 as measured baseline (before stress) and post-stress (n = 8 per each group)**

	Day 0 baseline	Day 0 post-stress	Day 21 baseline	Day 21 post-stress
Control	22.0 ± 2.64	101.9 ± 4.00	17.1 ± 2.47	85.6 ± 7.28
Olanzapine	22.0 ± 2.65	94.7 ± 6.54	16.9 ± 2.67	32.6 ± 4.42
*p*-values	1	0.452	0.973	0.001

*T*-test was used to compute the significance of differences between olanzapine treated and control groups.

**Table 2:** **Candidate genes of the dopamine pathway, showing significantly increased methylation** ***(p*** **< 0.01** ***)*** **following olanzapine treatment in hippocampus, cerebellum and liver, in a rat model** ***in vivo***

Gene name	Accession	Chr.	Strand	TSS	TTS	EH-CH	EC-CC	EL-CL
*Drd5*	NM_012768	chr14	-	77769487	77768059	1	1	1
*Camkk2*	NM_031338	chr12	+	34772493	34938479	1		1
*Calm3*	NM_012518	chr1	-	77252717	77245608	1		
*Kcnj2*	NM_017296	chr10	+	100574984	100576268	1		
*Kcnj4*	NM_053870	chr7	-	117603827	117601722	1		
*Cacna1c*	NM_012517	chr4	-	155517389	154895690	1		
*Ppp3ca*	NM_017041	chr2	+	234130175	234409232	1		1
*Adcy3*	NM_130779	chr6	+	27118324	27202275	1		
*Plcd1*	NM_017035	chr8	-	124052193	124023089	1		
*Ppp2r2d*	NM_144746	chr1	+	198640770	198674758	1		
*Ppp1r1b*	NM_138521	chr10	+	87121888	87131587	1		
*Nos1*	NM_052799	chr12	-	39869484	39811720	1		
*Itpr1*	NM_001007235	chr4	+	143705359	144030051	1		
*Ppp1r14b*	NM_172045	chr1	+	209648656	209650767	1		
*Crem*	NM_001110860	chr17	+	62770632	62837670	1		
*Slc18a2*	NM_013031	chr1	+	265789916	265824551		1	
*Ddc8*	NM_001017481	chr10	-	108349394	108336343		1	
*Drd2*	NM_012547	chr8	+	52641159	52707749			1
*Drd1a*	NM_012546	chr17	+	16655925	16658161			1

*Chr* chromosome, *TSS* transcription start site, *TTS* transcription termination site, *EH-CH, EC-CC, EL-CL* the number of differentially enriched peaks that showed increased in methylation in Olanzapine treated experimental (E) rats as compared to matched-controls (C) in hippocampus (H), cerebellum (C) and liver (L), respectively.

## Discussion

The significant increase in weight gain of olanzapine treated animals in the present study and in previously reported studies [18] has suggested that the paradigm adapted was capable of causing the metabolic disturbances observed in patients taking chronic olanzapine [19]. Furthermore, the significantly reduced locomotor activity of olanzapine treated rats indicated the therapeutic efficacy of the drug administered, which was comparable to the dosage applied in previous studies [20]. This argument was further supported by the observed changes in methylation of DA pathway genes.

Of special interest to this report are the genes involved in dopamine synthesis, transport, receptor, metabolism, interaction, and function [6]. The rationale for this focus stemmed from the fact that although antipsychotics interact with some dopamine receptors (D2), the actual mechanism of clinical effect behind antipsychotic efficacy in the treatment of psychosis is not fully understood. What is missing from the dopamine hypothesis of psychosis is the understanding of the underlying molecular mechanism(s) that may begin to reveal the full spectrum of the antipsychotics effects. Specifically, the results of the present study suggest an involvement of DNA methylation in genes of the dopamine pathway as an essential epigenetic mechanism in treating psychosis [21]. Our results showed that olanzapine causes an increase in DNA methylation in a significant (~40%) number of genes with an important role in dopamine neurotransmission. These include genes involved in the dopamine synthesis, transport, receptor, and metabolism (Figure 2). Further, the majority of DA pathway genes affected by olanzapine treatment were found to be hippocampus-specific, which is viewed as one of the primary sites for schizophrenia symptoms [21–23]. Also, a number of genes identified in the current study have been previously implicated in schizophrenia [24–27]. This increase in methylation in the DA pathway candidate genes is expected to interfere with transcription and suppress the functional gene product [28]. We also assessed if the methylated regions of individual genes have the potential to interfere with transcription. The results argue that gene-specific differentially methylated regions (DMRs) have necessary features of active promoters. All the affected DA pathway genes were differentially methylated in their promoter regions and therefore could result in altered gene expression [29]. For example, the promoter region of the *DRD5* gene is differentially methylated in all three tissues. This gene has been implicated in cognitive functions that include working memory [30]. Furthermore, *DRD1* and *DRD5* have been reported to have distinct regulatory roles on synaptic plasticity, spontaneous motor activity, memory and the information being processed by the hippocampus [31, 32]. Here we show that *DRD5*, which encodes the D5 subtype DA receptor, and has been previously described as a susceptibility gene for schizophrenia [33, 34], may result in diminished D5 subtype by increase in methylation following olanzapine treatment. This is expected in all three tissues studied. It is also known that *DRD5* interacts with *DRD2* in the process of augmenting or suppressing cellular functions [35]. Further, the *DRD5* gene region differentially methylated in response to olanzapine is compatible with methylation specific interference of transcription, as we have identified *in silico* predicted CTCF transcription factor binding sites (Figure 3) that warrant further confirmation. Similarly, the expression of *DRD2* could be regulated through methylation or demethylation of cytosines at the “putative” promoter region of *DRD2*[36–38]*.* Thus, the results included in this report offer a unifying mechanism of DNA methylation, which may represent the molecular basis for response to olanzapine, in a manner where the drug affects transcription of candidate genes from the dopamine pathway in addition to its effect on D2 blockade. This suggests that alterations to DNA methylation, in particular, and epigenetic changes, in general, may be used to develop novel strategies for the treatment of psychosis. We acknowledge the added value of confirming the methylation changes in the promoter regions of DA genes using an additional technique possibly involving a larger number of rats in our future study. Further, we intend to explore the expression of mRNA and proteins of relevant genes that are affected by DNA methylation so as to investigate the efficacy of olanzapine in treating psychosis via altering methylation status. Various confounding factors such as gene-diet/drug interactions could affect methylation changes [12, 39, 40]. Therefore, in the present study, proper caution was taken to exclude confounding factors that could possibly lead to methylation changes. For example, all experimental animals were kept in a uniform environment and were not exposed to other drugs or environments including diets, which may contribute to differences in methylation status of the treatment and control groups. All rats were of the same breed, sex, age and comparable body weight. They showed similar significant effects from olanzapine on weight gain and locomotor activity.

## Conclusions

The results of the present study suggest that chronic olanzapine may reduce DA activity in the long-term by altering gene methylation. Furthermore, olanzapine induced gene methylation may explain the delayed therapeutic effect of the drug, which occurs despite the rapid dopamine blockade and differential therapeutic responses of psychotic patients showing adequate D2 blockade.
